# Effect of the Combined Extracts of Herba Epimedii and Fructus Ligustri Lucidi on Sex Hormone Functional Levels in Osteoporosis Rats

**DOI:** 10.1155/2015/184802

**Published:** 2015-01-11

**Authors:** RenHui Liu, Xue Kang, LiPing Xu, HongLei Nian, XinWei Yang, HaoTian Shi, XiuJuan Wang

**Affiliations:** ^1^Beijing Key Lab of TCM Collateral Disease Theory Research, School of Traditional Chinese Medicine, Capital Medical University, No. 10 Xitoutiao, Youanmenwai, Fengtai District, Beijing 100069, China; ^2^Pharmacy of Traditional Chinese Medicine, Beijing Jishuitan Hospital, No. 10 Xinjiekou East Street, Xicheng District, Beijing 100035, China

## Abstract

The combination of Herba Epimedii and Fructus Ligustri Lucidi has been used to treat osteoporosis for almost 50 years by Professor Shizeng Li, a famous doctor of traditional Chinese medicine (TCM). However, it is unclear whether the combination of the effective constituents of the two herbs may have a protective influence on the skeleton. In the present study, we investigated the effects of the combination extracts of Herba Epimedii and Fructus Ligustri Lucidi on rat model of osteoporosis induced by retinoic acid by gavage. With administrations of the combination extracts of the two herbs (50, 100, and 200 mg/kg/day) via oral gavage for 3 weeks, bone mineral density (BMD), femur histomorphometry, some sex hormones, and sex hormone receptors were measured. Results showed that the combined extracts could increase BMD, affect bone histomorphometry, coordinate the sex hormones at the level of hypothalamus-pituitary-gonad axis, and increase the protein and mRNA expressions of sex hormone receptors. The findings suggested that the combination extracts of Herba Epimedii and Fructus Ligustri Lucidi might be beneficial as an alternative medicine for the prevention and treatment of osteoporosis.

## 1. Introduction

Osteoporosis is one kind of the most common bone remodeling diseases characterized by reduction of bone mass and microstructural deterioration of bone tissue. The essence of this disease is caused by an imbalance of bone remodeling, that is, less bone formation and more bone resorption which may ultimately result in skeletal fragility, an increased risk of hip and vertebral fracture [1]. It mainly affects postmenopausal women and elderly men and has already become one of the most serious threats for health [2, 3]. It is estimated that, worldwide, over 200 million people suffer from osteoporosis, and the annual attendant costs have exceeded approximately 10 billion dollars for this disease [4]. It is well known that bone is an endocrine tissue that is sensitive to androgens and estrogens, and sex steroids play an important role in the maintenance of bone health. Low levels of estrogen and androgen were found to be associated with high bone turnover, low bone mineral density (BMD), and high risk of osteoporotic fractures in both men and women [5]. Hormone replacement therapy (HRT) has been proven to be efficacious in preventing bone loss and reducing the incidence of skeletal fractures in postmenopausal women and elderly men [6, 7]. However, long-term HRT increases the high risk of breast cancer, coronary heart disease, thromboembolic events, and vaginal bleeding [8, 9]. Concerns about the adverse effects of HRT have led to interest in the antiosteoporotic activity of natural products.

According to the theories of TCM, bone health is considered to be closely related to kidney function, and “kidney deficiency” is thought to be the root of all pathologies related to bones and joints. Thus, the treatment of osteoporosis accordingly follows the principle of strengthening the kidney function. Herba Epimedii (*Yinyanghuo*) and Fructus Ligustri Lucidi (*Nvzhenzi*) documented in Chinese ancient medicinal literatures have strong actions of replenishing kidney-*yang* and kidney-*yin,* respectively. So they have been used to strengthen bone and treat osteoporosis for a long time in China. Considering osteoporosis is a chronic and complex disease with kidney-deficiency syndrome that should be treated with long-term medication intervention, both* Yang*-tonifying prescription with the property of warm dryness and* yin*-tonifying prescription with the sticky and greasy property are unsuitable for long-term clinical application. According to TCM theories,* Yang*-tonifying herbs matched with adequate* Yin*-tonifying herbs can make living things freely grow, flourish, and eliminate the side effects of each other. Professor Shizeng Li, a famous doctor of TCM, has used the decoction combination of Herba Epimedii and Fructus Ligustri Lucidi to treat osteoporosis for almost 50 years. In our previous researches, it was demonstrated that the combination of Herba Epimedii and Fructus Ligustri Lucidi could reverse the collagen type I and bone metabolism abnormality, associated with increasing BMD and biomechanical properties, as well as improving pathological condition of the bone tissue in rats with osteoporosis induced by retinoic acid [10, 11]. The effect of combined therapy with Herba Epimedii and Fructus Ligustri Lucidi was better than one alone. Up to now, many studies had confirmed the antiosteoporotic effect of the total flavonoids of Herba Epimedii [12–15] and the total iridoid and flavonoids extracted from Fructus Ligustri Lucidi [16–18].

Researchers have found that hypervitaminosis A causes accelerated bone resorption, bone fragility, and spontaneous fractures [19]. Furthermore, high intake of dietary vitamin A has been associated with reduced BMD and increased risk for hip fractures in humans [20]. We decided to conduct experiments with an osteoporosis rat model induced by retinoic acid. Because of similarities in skeletal responses to estrogen deficiency, typicalness of bone microstructure, and reversibility after administration of medicine, retinoic acid-induced osteoporosis model is considered as a convenient and standard model for the investigation of pharmacological changes after treatments for osteoporosis. In the present study, the antiosteoporosis effect of the combined extracts of Herba Epimedii and Fructus Ligustri Lucidi was investigated. In addition, no matter how complex ingredients and action mechanisms of the herbal preparations, but the phytohormones such as phytoestrogens and phytoestrogens could be playing an important role in the amelioration of bone loss [21–23]. Considering that the periodicity and estrus of female rat affect the peak in blood estradiol levels, male rats were selected for our experimental study in order to avoid interindividual differences in hormone levels.

## 2. Material and Methods

### 2.1. Preparation of Herbal Extracts

Herba Epimedii, the dried leaf of* Epimediium brevicornum Maxim.,* and Fructus Ligustri Lucidi, the dried mature seed of* Ligustrum lucidum Ait.*, were purchased from Beijing* Tongrentang* pharmaceutical Co. Ltd., China, and authenticated by an expert herbalist at Capital Medical University. They were stored in a dry and sealed container at 4°C.

For the preparation of extracts, Herba Epimedii (1000 g) was extracted three times with 90% ethanol (10 000 mL) for 3 h, 2 h, and 2 h at 70°C in a reflux apparatus. The extracts were mixed, filtered, and concentrated under reduced pressure, until recovery to no alcohol precipitation. Then with petroleum ether extraction three times to remove chlorophyll, the extracts were washed with D-101 macroporous resin to colorless, eluted with 90% ethanol, and steamed to yield a dark yellow powder, namely, total flavonoids of Herba Epimedii (TFE). The yield of Herba Epimedii extract was 2.5%. Based on the phytochemical test (Pharmacopoeia of the People's Republic of China, 2010 Edition), the content of TFE was 80% calculated by Icariin. Fructus Ligustri Lucidi (1000 g) was extracted three times with 75% ethanol (10 000 mL) for 3 h, 2 h, and 2 h at 70°C in a reflux apparatus. The extracts were mixed, filtered, and concentrated under reduced pressure, until recovery to no alcohol precipitation. Using AB-8 macroporous resin, the extracts were washed with distilled water and then with 75% ethanol after the water solution was discarded. The steamed extracts are total iridoid and flavonoids of Fructus Ligustri Lucidi (TIFL). The yield of Fructus Ligustri Lucidi extract was 5%. Based on the phytochemical test (Pharmacopoeia of the People's Republic of China, 2010 Edition), the content of TIFL was more than 80% calculated by Oleanolic Acid and Rutin, respectively.

The extract combination of Herba Epimedii and Fructus Ligustri Lucidi was composed of TFE and TIFL with a ratio of 2 to 3 (equivalent to the raw herbs ratio of 4 to 3). Doses of Herba Epimedii and Fructus Ligustri Lucidi were chosen based on the clinical practice by Shizeng Li and our previous studies, which have demonstrated that combination of the two herbs has the effects on the prevention and treatment of osteoporosis [10, 11, 24]. Before application, TFE and TIFL were mixed with the required amount in the ratio of 2 to 3. The mixture was dissolved in deionised water with concentrations of 5 mg/mL, 10 mg/mL, and 20 mg/mL, respectively.

### 2.2. Animals

Fifty male Wistar rats, weighing 230 to 270 g with the average age of three months, were purchased from Vital River Laboratory Animal Technology Co. Ltd. (Beijing, China). The experiment complied with the Animal Management Rule of the Ministry of Public Health, China, and the experimental protocol was approved by the Animal Care Committee of Capital Medical University, Beijing, China. All the animals were cared for in the Experimental Animal Center of Capital Medical University. During the whole experiment, the animals were housed in stainless cages (three rats per cage) at conventional controlled conditions (temperature of 23 ± 2°C, relative humidity of 50 ± 10%, 12-hour light-dark cycle). They were allowed for free access to the standard laboratory food and tap water.

### 2.3. Experimental Protocol

The rats were acclimated to conditions for one week before the experiment and randomly divided into 5 groups of 10 rats each, including normal control group, model control group, and three treatment groups [Extract-L (50 mg/kg), Extract-M (100 mg/kg), and Extract-H (200 mg/kg)]. All rats received intragastric administration of retinoic acid (70 mg/kg) daily for two weeks to establish osteoporosis model except those of normal control group. Rats in treatment groups were administered with the combined extracts via oral gavage at the dose of 50, 100, and 200 mg/kg body weight, respectively. At the same time, the rats in the normal control group and model control group were given the same volume of distilled water. Three weeks later, rats were anesthetized with ethyl carbamate (4 mL/kg, ip). Blood samples were collected and separated simultaneously using a centrifuge (Biofuge 15R, Heraeus Sepatech, USA), and serum was collected finally and stored at −80°C prior to assay. Each of the rats had the bilateral femurs and the left tibia dissected out and cleaned of any tissues excised. Each left femur was submerged in 4% neutral-buffered paraformaldehyde solution in PBS (pH 7.4) for 24 h and decalcified in a solution of 1% ethylene diamine tetraacetic acid solution (EDTA; PH 7.36) for 6 weeks. After that, each left femur sample was cut along the coronal plane, embedded in paraffin, and cut longitudinally into 5 *μ*m sections by freezing microtome (OM2563, TBS, CA) for histomorphometry and immunohistochemistry. Each right femur was wrapped with gauze dipped in physiological saline for BMD test. Each left tibia was frozen by liquid nitrogen for Real-Time Polymerase Chain Reaction (PCR) test.

### 2.4. BMD Measurement

The BMD of each right femur were measured* ex vivo* by a dual-energy X-ray absorptiometer (DXA; Lexxos-2000; Medlink, France) equipped with appropriate software for bone assessment in small animals [25]. The scanning position included the whole femur, femur head, and femur neck. The scan resolution was 1.0 × 1.0 mm and scan speed was 10 mm/s. The measurements with repositioning of the bones were repeated for three times to calculate the coefficients of variation (CV). The CV for BMD on the whole femur, femur head, and femur neck were 2.3%, 1.9%, and 2.1%, respectively. The results of the BMD in the whole femur, femur head, and femur neck were expressed as the mean value.

### 2.5. Bone Histomorphometry

Staining with hematoxylin and eosin (HE), each left femur section was observed for microarchitectural changes under a microscope (ECLIPSE 80i, Nikon, Japan), especially the structure and morphology of trabecular bone. Histomorphometry variables were analyzed in trabecular bone using an image analyzing computer system (NIS-Elements BR 3.2, Nikon, Japan) linked to a microscope. The parameters measured included trabecular bone area (Tb.Ar), trabecular thickness (Tb.Th), trabecular number (Tb.N), and trabecular separation (Tb.Sp). All of the histomorphometric indices were reported according to the standardized nomenclature recommended by the American Society of Bone and Mineral Research Histomorphometry Nomenclature Committee [26]. The data were obtained by blind measurements.

### 2.6. Serum Gonadal Hormones' Levels

The serum levels of estradiol (E_2_), testosterone (T), luteinizing hormone (LH), follicle stimulating hormone (FSH), and gonadotropin-releasing hormone (GnRH) were determined. Serums E_2_, T, LH, and FSH were measured by rat E_2_, T, and LH radioimmunoassay kits (Sino-UK Institute of Biological Technology, Beijing, China) for control, standard, and duplicate tests. Serum GnRH was measured using a rat GnRH ELISA kit (BlueGene Biotech Co. Ltd., Shanghai, China) by enzyme-linked immunosorbent assay (Immunodiagnostic System Ltd., Boldon, UK), and absorbance was read using ELISA reader (Thermo, USA) at 450 nm.

### 2.7. Immunohistochemical Analysis

Frozen sections were mounted on glass slides and used for immunohistochemical assessment. Primary antibodies [anti-rat androgen receptor (AR, 1 : 100) and anti-rat estrogen receptor (ER, 1 : 100)] were purchased from Santa Cruz Biotechnology (Santa Cruz, CA, USA). The tissue slides were rinsed in PBS and incubated with the primary antibodies for 1 h at 37°C and then rinsed with PBS three times for 3 min. The slides were then incubated with the appropriated biotinylated antibody (EnVision, Dako, DEN) for 30 min at 37°C and then rinsed with PBS three times for 3 min, followed by incubation with a solution containing 3,3-diaminobenzidine and hydrogen peroxide at room temperature for 30 s, and then rinsed in running water. After that, the slides were counterstained with Harris hematoxylin and sealed for microscopic analysis. As negative control, nonimmune goat serum instead of the primary antibody was served as negative control. All measurements were performed with the Nikon ECLIPSE 80i biomicroscope and NIS-Elements BR 3.2 image analysis system (Nikon, Japanese). Five random images within tibiae from two sections were taken and further analyzed by using zoomed-in field at 400x magnification. We measured the integral optical density of AR or ER-positive cells under each examined field for each section and calculated the average number as the final result of this sample.

### 2.8. Quantitative Real-Time PCR (qPCR)

Total RNA was isolated from each left tibia using TRIzol reagent (Invitrogen, CA, USA) according to the manufacturer's recommendations. Total RNA (2 *μ*g) was reverse-transcribed using the Superscript First Strand Synthesis System (Invitrogen, CA, USA) to generate complementary DNA (cDNA). The qPCR amplification was performed using the SYBR-green detection of PCR products in real time with an ABI-7500 Sequence Detection System (Applied Biosystems, Foster City, CA, USA). The primers used in the qPCR analysis are presented in Table 1. The PCR program was performed for 40 cycles with each cycle consisting of 30 s of denaturation at 95°C, 1 min of annealing at 95°C, and 1 min of extension at 68°C. Gene expression was quantified by means of the comparative Ct method (ΔΔCt) and the relative quantification (RQ) was calculated as 2^−ΔΔCt^. Relative mRNA levels of ER and AR were examined and normalized to *β*-actin mRNA expression in each sample. The melting curves for each PCR reaction were generated to ensure the purity of the amplification product. A no-template negative control was included in each experiment.

### 2.9. Statistical Analysis

Results of all measurements were presented as means ± standard deviation (SD). The data analysis was performed using the SPSS 13.0 (SPSS Inc., Chicago, USA). All of the data were tested for normality, using the Kolmogorov-Smirnov test, and passed. A one-way analysis of variance (ANOVA) was performed to determine whether there were statistically significant differences (*P* < 0.05) among the experimental groups. Duncan's multiple range post hoc test was used for comparisons between individual groups and to determine which means differed statistically significantly (*P* < 0.05). Pearson Correlation Analysis was used for detecting the correlation of data.

## 3. Results

### 3.1. Effects of the Combination with TFE and TIFL on BMD

**Figure 1:**
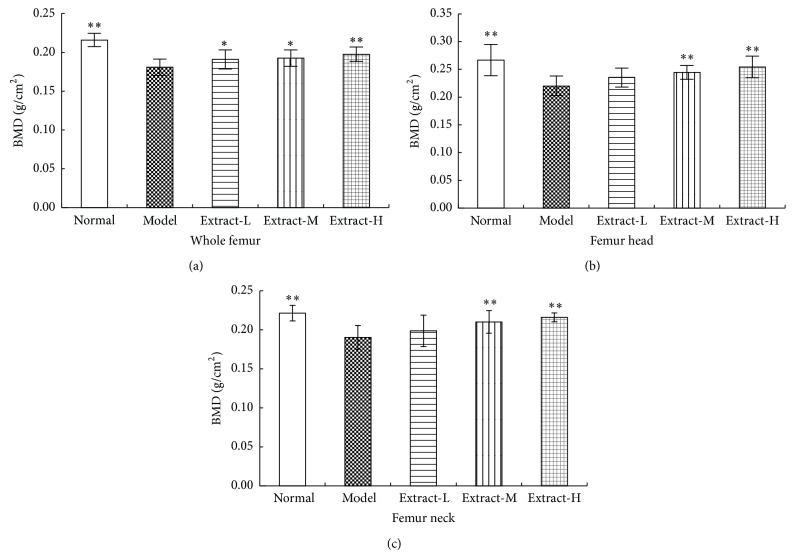
Effects of the combination with TFE and TIFL on BMD in whole of femur (a), femur head (b), and femur neck (c) in osteoporosis rats. BMD of the whole femur, femur head, and femur neck in osteoporosis rats was significantly lower than that in normal group. Mean ± SD, *n* = 10. ^∗^ *P* < 0.05 and ^∗∗^ *P* < 0.01 versus model group.

To investigate whether the combination with TFE and TIFL has antiosteoporotic effects, the BMD of different parts of femur were measured by DXA.  Figure 1 showed that BMD levels of the whole femur, femur head, and femur neck were all significantly reduced in osteoporosis rats (all *P* < 0.01). Treatment with either medium dose or high dose of the combination with TFE and TIFL for 3 weeks significantly increased the BMD levels of the whole femur, femur head, and femur neck, compared to the model group (*P* < 0.05 or *P* < 0.01), while low dose group only had a remarkable effect on the whole femur (*P* < 0.05).

### 3.2. Effects of the Combination with TFE and TIFL on Bone Histomorphometry

**Figure 2:**
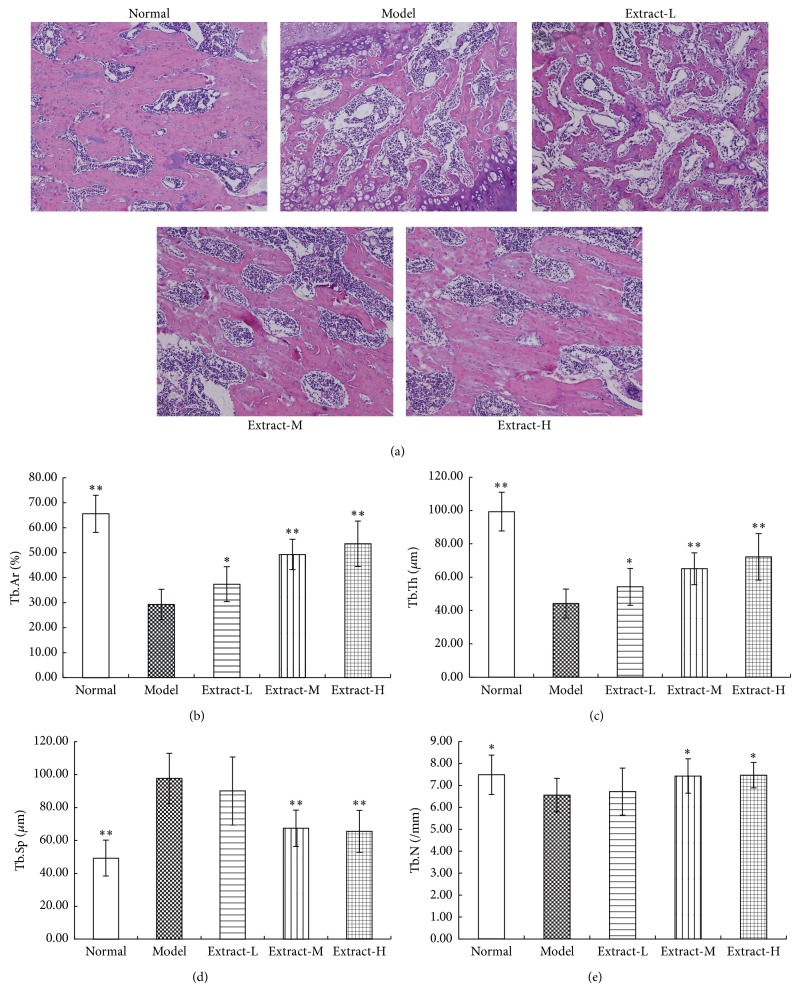
Effects of the combination with TFE and TIFL on bone histomorphometry in osteoporosis rats. (a) Representative microscope images of trabecular bone microarchitecture in femurs (HE, ×100). Bone histomorphometry of the trabecular bone included Tb.Ar (b), Tb.Th (c), Tb.Sp (d), and Tb.N (e). Mean ± SD, *n* = 10. ^∗^ *P* < 0.05 and ^∗∗^ *P* < 0.01 versus model group.

HE staining showed that cancellous bone was formed of a dense network of trabecular bone in the normal group and osteocytes appeared in their lacunae. The endosteal surface of trabecular bone was lined by osteoprogenitor cells, osteoblasts, and osteoclasts in Howship's lacunae. Bone marrow spaces were seen between the trabeculae. The results in model group revealed that the cancellous bone lost its normal architecture and trabecular bone became thinner and discontinuous, and osteoclasts apparently increased as compared with the normal group. After administration of the combination with TFE and TIFL for 3 weeks, pathological morphology of trabecular bone was improved significantly. The effects of medium dose and high dose group were more obvious. The cancellous bone partially regained near normal structure and the trabecular bone widened, its broken points lessened, and its Howship's lacunae shallowed as compared to the normal group (Figure 2(a)). Bone histomorphometry such as Tb.Ar (Figure 2(b)), Tb.Th (Figure 2(c)), and Tb.N (Figure 2(d)) in model group was decreased (*P* < 0.05 or 0.01), and Tb.Sp (Figure 2(e)) was increased (*P* < 0.01). Medium dose and high dose group had remarkable effect on each index (*P* < 0.05 or 0.01), while low dose group only had a remarkable effect on Tb.Ar% and Tb.Th (all *P* < 0.05), compared with the osteoporosis rats without treatment.

### 3.3. Effects of the Combination with TFE and TIFL on Serum Levels of Gonadal Hormones

**Figure 3:**
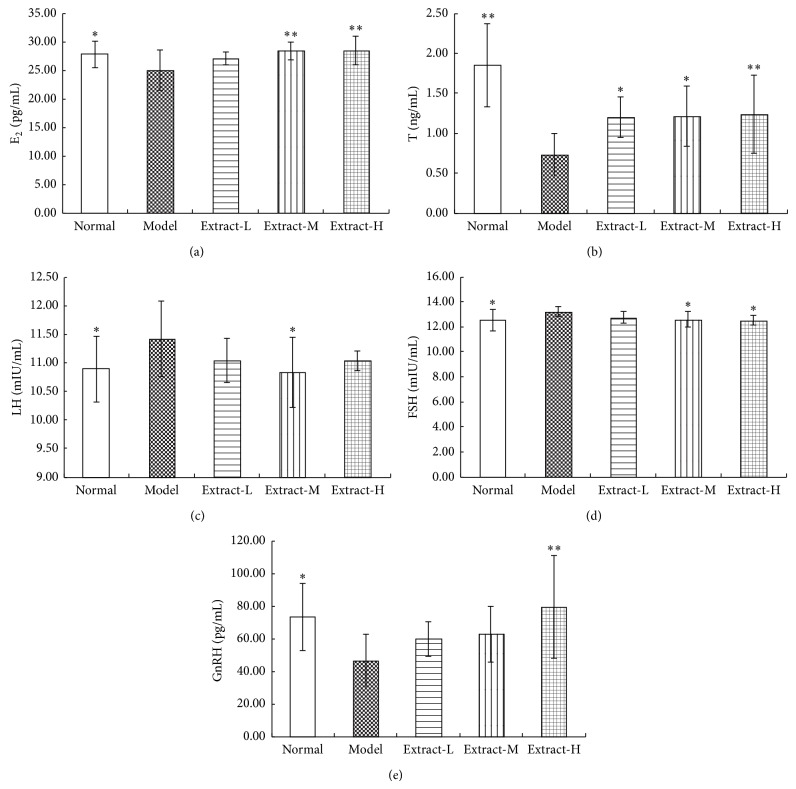
Effects of combination with TFE and TIFL on serum levels of gonadal hormones in osteoporosis rats. Gonadal hormones E_2_ (a), T (b), LH (c), FSH (d), and GnRH (e) were shown in (a), (b), (c), (d), and (e), respectively. Mean ± SD, *n* = 10. ^∗^ *P* < 0.05 and ^∗∗^ *P* < 0.01 versus model group.

As shown in Figure 3, the content of serum E_2_ and T was significantly decreased after modeling (*P* < 0.05 or *P* < 0.01). Compared with the model group, serum T content in each administration group was remarkably increased (*P* < 0.05 or *P* < 0.01), and E_2_ content in medium and high dose group was significantly increased (*P* < 0.05 or *P* < 0.01). Compared with the normal control group, serum LH and serum FSH content were increased significantly, while the content of GnRH was significantly reduced in osteoporosis rats induced by retinoic acid (all *P* < 0.05). After administration of the combination with TFE and TIFL for 3 weeks, serum LH content was reduced significantly only in medium dose group (*P* < 0.05), the content of FSH in medium and high dose group was decreased significantly (*P* < 0.05), and serum GnRH level was increased remarkably in high dose group (*P* < 0.01).

### 3.4. Effects of the Combination with TFE and TIFL on Expressions of Sex Hormone Receptors

**Figure 4:**
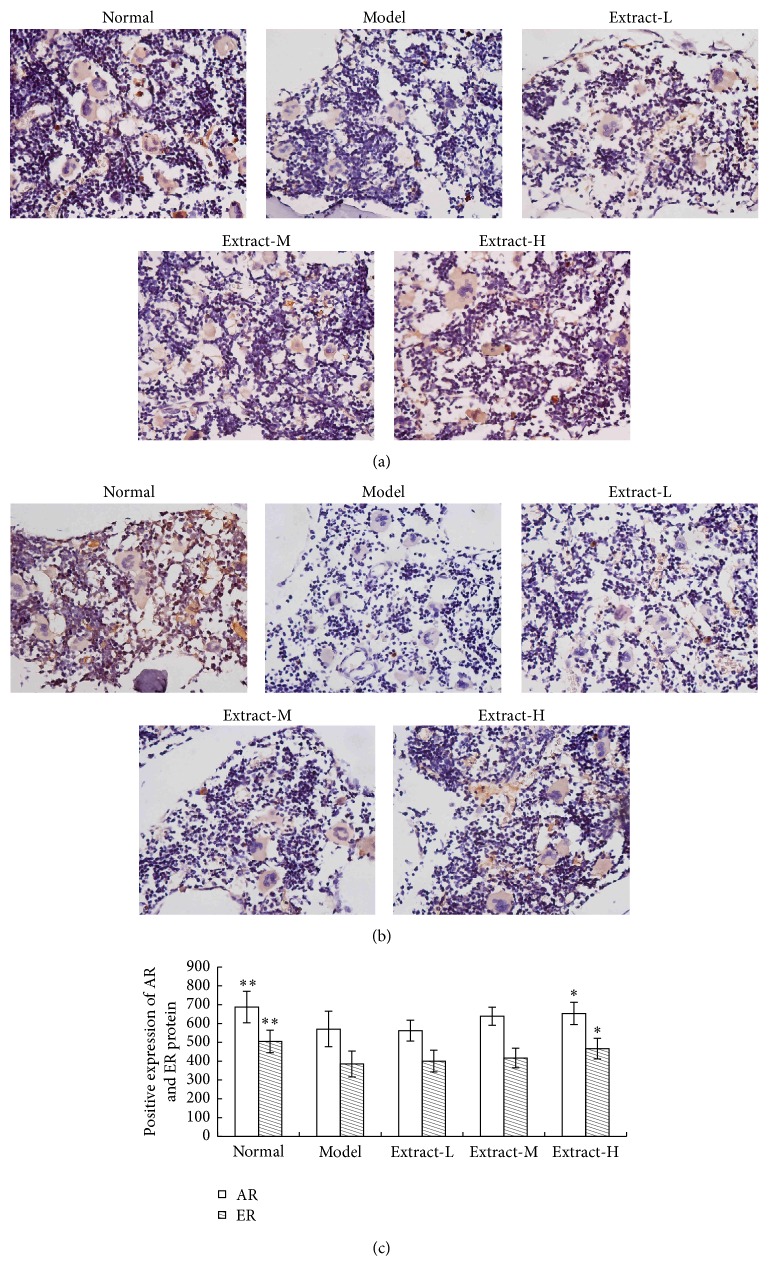
Effects of the combination with TFE and TIFL on protein expressions of AR and ER in osteoporosis rats. Expression levels of AR and ER were estimated by immunohistochemical analysis. AR protein expression (a), ER protein expression (b), and quantitative analysis of protein expressions of AR and ER (c). Mean ± SD, *n* = 7. ^∗^ *P* < 0.05 and ^∗∗^ *P* < 0.01 versus model group.

Immunohistochemical staining results showed that positive expressions of AR and ER protein in femur were located in cytoplasm and dyed pale brown. Positive areas are mainly located in bone marrow stroma. Proteins of AR and ER were strongly expressed in normal control group, while the two receptors were weakly expressed in osteoporosis rats. The level of AR or ER positive expression in each administration group was situated between the normal group and model group (Figures 4(a) and 4(b)). Quantitative analysis showed that osteoporosis caused a significant decrease in protein expressions of AR and ER in femur compared to normal control group (*P* < 0.01). Treatment of the combination with TFE and TIFL for 3 weeks increased the protein expressions of AR and ER in high dose group (*P* < 0.05) (Figure 4(c)).

**Figure 5:**
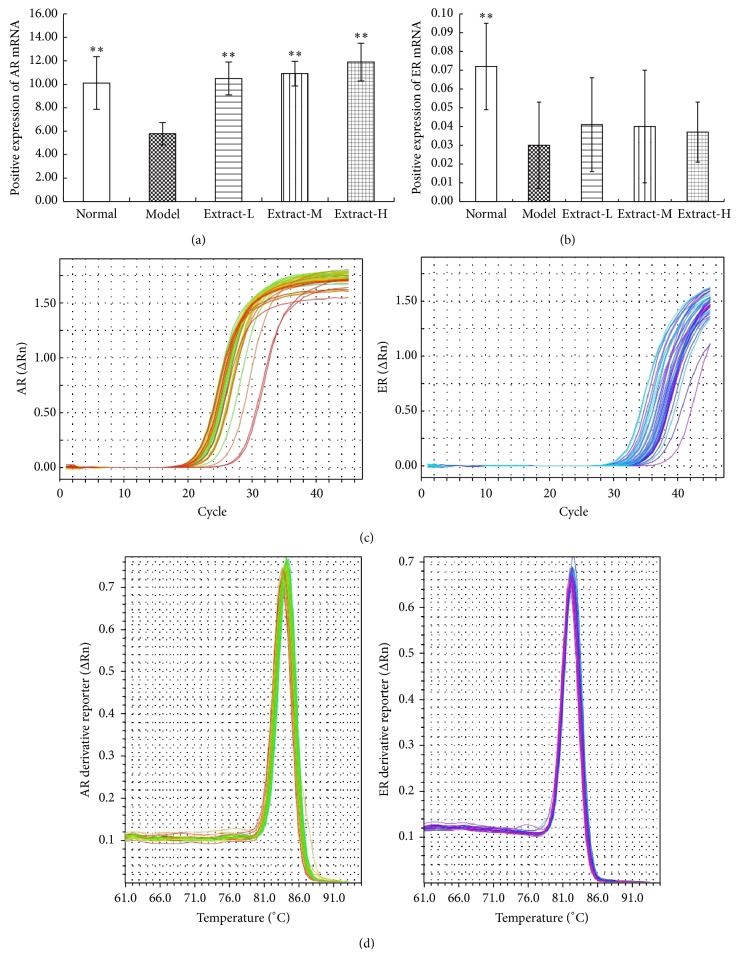
Effects of combination with TFE and TIFL on mRNA expressions of AR and ER in osteoporosis rats. (a) AR mRNA expression was determined by qPCR with β-actin as an internal control. (b) ER mRNA expression was determined by qPCR with β-actin as an internal control. (c) Amplification curves of the mRNA expressions of AR and ER. (d) Melt curves of the mRNA expressions of AR and ER. The mRNA expressions of AR and ER in osteoporosis rats were significantly lower than that in normal group. Mean ± SD, *n* = 6. ^∗^ *P* < 0.05 and ^∗∗^ *P* < 0.01 versus model group.

To further understand whether the combination with TFE and TIFL affect the mRNA levels of ER and AR, we analyzed mRNA expression levels of ER and AR in tibia of rats using qPCR. The mRNA expressions of AR and ER in the model group were significantly alleviated compared to the normal control group (*P* < 0.01). Treatment with the combination with TFE and TIFL for 3 weeks could increase significantly AR mRNA expression (all *P* < 0.01) but had no remarkable effect on ER mRNA expression (Figure 5).

### 3.5. Analysis of the Correlation within Sex Hormones and Sex Hormone Receptors

**Figure 6:**
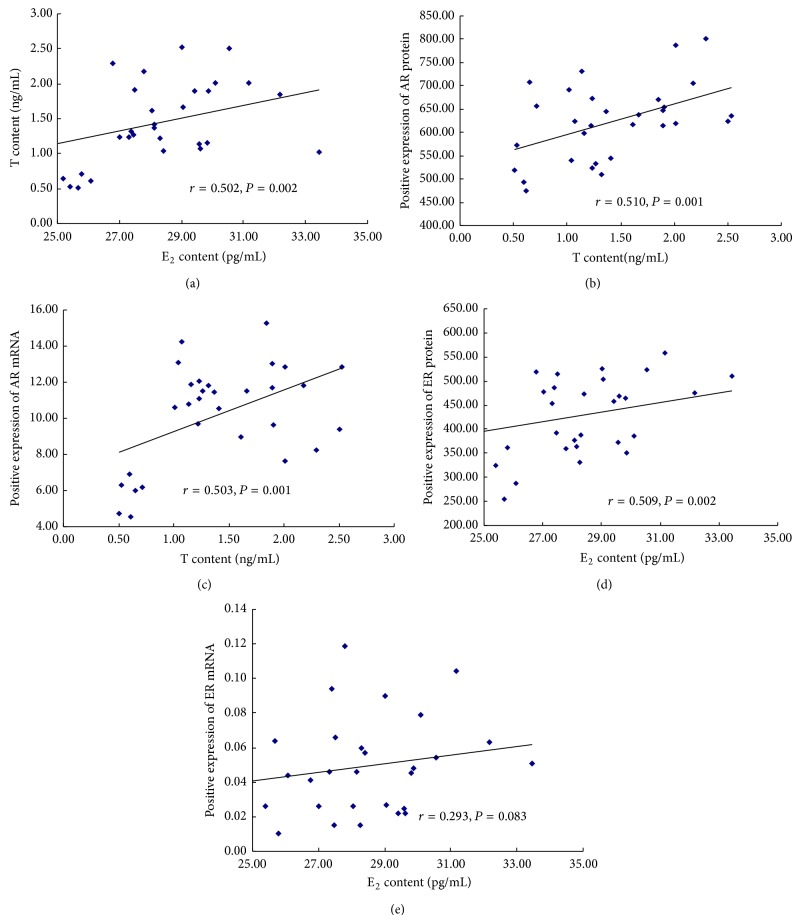
Analysis of the correlation within sex hormones and sex hormone receptors. (a) The correlation between serum content of T and E_2_; (b) the correlation between serum T content and AR protein expression of femur; (c) the correlation between serum T content and AR mRNA expression of tibia; (d) the correlation between serum E_2_ content and ER protein expression of femur; (e) the correlation between serum E_2_ content and ER mRNA expression of tibia.

To explore whether there were direct responses between E_2_ and T and between sex hormones and corresponding sex hormone receptors, we conducted a correlation analysis and the results were shown in Figure 6. There were significantly positive correlations between T and E_2_ content in serum (*r* = 0.502, *P* = 0.002), between serum T content and AR protein expression of femur (*r* = 0.510, *P* = 0.001), between serum T content and AR mRNA expression of tibia (*r* = 0.503, *P* = 0.001), and between serum E_2_ content and ER protein expression of femur (*r* = 0.509, *P* = 0.002).

## 4. Discussion

The kidney-tonifying Chinese herbal medicines, Herba Epimedii and Fructus Ligustri Lucidi, have been widely used to treat osteoarticular disease for thousands of years in China. Professor Shizeng Li, a famous doctor of TCM, has used the decoction compatibility of Herba Epimedii (as a kidney-*yang* tonifying herb) and Fructus Ligustri Lucidi (as a kidney-*yin* tonifying herb) to treat osteoporosis for almost 50 years. Our previous studies have confirmed that the compatibility of Herba Epimedii and Fructus Ligustri Lucidi have antiosteoporosis effects on the asthmatic rats treated with dexamethasone [27], glucocorticoid-induced osteoporosis rats [24, 28], and retinoic acid-induced osteoporosis rats [10, 11]. These results suggest that the two herbs have a good application prospect for the prevention and treatment of osteoporosis. Thus, the two herbal medicines will undoubtedly continue to be used as a cost-effective alternative to treating osteoporosis and commercial pharmaceutical products by the doctor of TCM. However, the international medical community only accepts herbal extracts, not herbs, as an alternative therapeutic regime for the management of osteoporosis. So we chose total flavonoids of Herba Epimedii (TFE) and total iridoid and flavonoids of Fructus Ligustri Lucidi (TIFL) as the two herbs' active components to evaluate the antiosteoporosis effects in this study.

BMD and bone histomorphometry were used to evaluate the pharmacodynamics effects of the combination with TFE and TIFL on osteoporosis rats induced by retinoic acid. BMD has been known as the main contributor to bone quality and a surrogate measure of bone strength [29]. In this study, BMD levels of the whole femur, femur head, and femur neck in the model group were all significantly reduced. BMD is markedly decreased probably due to an increase in bone turnover with excess resorption relative to formation in the osteoporosis rats compared to the normal rats. Treated with either medium dose or high dose of the combination with TFE and TIFL, rat femur BMD significantly increased compared to the model rats. Although BMD is considered as an important determinant of bone strength, it does not take into account bone surface remodeling activity occurring in trabecular bone. In order to observe the changes of bone surface remodeling activity, we had used indices of bone histomorphometry to further explain the change of BMD. The trabecular bone microarchitecture is universally considered to be a good predictor of bone mass loss and bone structure deterioration [30]. Because the metaphyseal region of the distal femur was the main growth plate of trabecular bone, we evaluated the pathological changes of this region. According to the results of our experiment, retinoic acid induced significant femoral osteopenia as shown by decreasing Tb.Ar%, Tb.Th, and Tb.N as well as increasing Tb.Sp, which indicated that osteopenia and poor bone strength had formed. Further observation suggested the combination with TFE and TIFL had significant effects on trabecular microarchitectural properties, which were reflected by the increase in Tb.Ar%, Tb.Th, and Tb.N as well as the decrease of Tb.Sp. This research results indicated that treatment with the combination with TFE and TIFL significantly reduced bone loss and enhanced bone strength in the osteoporosis rats induced by retinoic acid.

Sex hormones are an important regulator for the occurrence of osteoporosis. Androgen reduction was associated with bone loss not only in postmenopausal but also in male osteoporosis [31]. Serum T content deficiency, which led to BMD and bone quality decreased, was an important cause of male incidence of osteoporosis [32, 33]. Generally speaking, estrogen played a leading role in the regulation of bone resorption. Estrogen level in the elderly male patients was the only decisive index correlated with BMD and bone resorption [34]. In this study, we found that retinoic acid could attenuate the serum levels of T and E_2_ compared with normal control group, while the combination of TFE and TIFL could significantly increase sex hormone level in osteoporosis rats. The results suggested that the combination of TFE and TIFL could play a role in antiosteoporosis by raising the levels of sex hormones. Furthermore, there was a positive relationship between the level of serum estrogen and androgen.

Osteoporosis was induced by not only a certain hormone change but also the entire hypothalamic-pituitary-adrenal axis at each level [35]. Thus, either the dysfunction of hypothalamic-pituitary-gonadal axis or the secretion disorder of each level of hormone is very likely to lead to osteoporosis. The synthesis of sex steroids is under hypothalamic-pituitary feedback control via gonadotropins (FSH and LH). FSH is a physiological stimulating factor of bone absorption; it can stimulate bone resorption and directly involve in the regulation of bone mass and bone metabolism by Tumor Necrosis Factor-*α* (TNF-*α*) [36]. Serum FSH and LH levels are negatively correlated with BMD and increased levels of FSH and LH can lead to increased risk of osteoporosis [37]. Simultaneously, the sex hormone deficiency caused by the elevated levels of FSH and LH can restrain the secretion of GnRH produced by the hypothalamus. In this study, the results showed that the content of FSH and LH significantly increased, while GnRH significantly decreased due to a lack of sex hormone, suggesting that low sex hormone levels and disorder of the gonadal hormone secretion were one of the important causes of abnormal bone metabolism. the combination with TFE and TIFL may significantly prevent increased levels of FSH and LH induced by sex hormone deficiency and stimulate the secretion of GnRH, which indicated that regulation of related hormones at each level of hypothalamic-pituitary-gonad axis could be one of the pharmacological mechanisms of the combination with TFE and TIFL for osteoporosis treatment.

Sex hormone can start the response and regulate the bone remodeling only binding with the corresponding receptor. Studies suggest that both AR and ER are required for optimal cortical bone expansion via actions in immature osteoblasts and trabecular bone maintenance via actions in more differentiated osteoblasts and osteocytes [38–40]. Our results showed that protein and mRNA expressions of both AR and ER in bone tissue were significantly decreased in osteoporosis reduced by retinoic acid, which suggested that retinoic acid not only leads to the low level of sex hormone but also induces downregulation of the expressions of sex hormone receptors, which may be related to responsiveness to sex hormone decreased. The combination of TFE and TIFL could reverse this effect induced by retinoic acid and significantly upregulate protein or mRNA expressions of AR and ER. The further correlation analysis showed the serum level of T was positively correlated with protein or mRNA expression of AR and the serum level of E_2_ was significantly correlated with protein expression of ER. The results indicated that expressions of sex hormone receptors would be up (or down) accompanied by increased (or decreased) sex hormone levels. There was a direct response between sex hormone receptors and sex hormones, and the transcription activity levels after the combination with a hormone and its receptor could be an important factor that affects the occurrence of osteoporosis.

## 5. Conclusions

This study demonstrated that the combination with TFE and TIFL had potential protective effects on osteoporosis rats induced by retinoic acid. The combination with TFE and TIFL is able to increase BMD, reverse pathological changes of bone tissue, coordinate hormones of the hypothalamic-pituitary-gonadal axis, and upregulate the expression of sex hormone receptors. The present data suggest that the combination with TFE and TIFL may be a reasonable natural alternative for the prevention and treatment of osteoporosis. However, further detailed mechanistic investigation of the antiosteoporotic effects of the combination with TFE and TIFL on bone metabolism is required.

## Figures and Tables

**Table 1:** Primers used for qPCR analysis.

Primer	Forward primer	Reverse primer
rno-AR	5′-ggcaaaggcactgaagagac-3′	3′-cccagagctacctgcttcac-5′
rno-ER	5′-tccggcacatgagtaacaaa-3′	3′-tgaagacgatgagcatccag-5′
β-actin	5′-agccatgtacgtagccatcc-3′	3′-accctcatagatgggcacag-5′

## Abbreviations

TCM: Traditional Chinese medicine
HRT: Hormone replacement therapy
BMD: Bone mineral density
TFE: Total flavonoids of Herba Epimedii
TIFL: Total iridoid and flavonoids of Fructus Ligustri Lucidi
DXA: Dual-energy X-ray absorptiometer
Tb.Ar: Trabecular bone area
Tb.Th: Trabecular thickness
Tb.N: Trabecular number
Tb.Sp: Trabecular separation
E_2_: Estradiol
T: Testosterone
LH: Luteinizing hormone
FSH: Follicle stimulating hormone
GnRH: Gonadotropin-releasing hormone
AR: Androgen receptor
ER: Estrogen receptor.
